# Terahertz Modulation and Ultrafast Characteristic of Two-Dimensional Lead Halide Perovskites

**DOI:** 10.3390/nano12203559

**Published:** 2022-10-11

**Authors:** Hongyuan Liu, Xunjun He, Jie Ren, Jiuxing Jiang, Yongtao Yao, Guangjun Lu

**Affiliations:** 1 School of Computer Science, Harbin University of Science and Technology, Harbin 150080, China; 2 Key Laboratory of Engineering Dielectric and Applications (Ministry of Education), School of Electrical and Electronic Engineering, Harbin University of Science and Technology, Harbin 150080, China; 3 College of Science, Harbin University of Science and Technology, Harbin 150080, China; 4 National Key Laboratory of Science and Technology on Advanced Composites in Special Environments, Harbin Institute of Technology, Harbin 150080, China; 5 School of Electronic and Information Engineering/School of Integrated Circuits, Guangxi Normal University, Guilin 541004, China

**Keywords:** 2D R-P type perovskite, OPTP, terahertz modulation, ultrafast characteristics, Drude-Smith model

## Abstract

In recent years, two-dimensional (2D) halide perovskites have been widely used in solar cells and photoelectric devices due to their excellent photoelectric properties and high environmental stability. However, the terahertz (THz) and ultrafast responses of the 2D halide perovskites are seldom studied, limiting the developments and applications of tunable terahertz devices based on 2D perovskites. Here, 2D R-P type (PEA)_2_(MA)_2_Pb_3_I_10_ perovskite films are fabricated on quartz substrates by a one-step spin-coating process to study their THz and ultrafast characteristics. Based on our homemade ultrafast optical pump–THz probe (OPTP) system, the 2D perovskite film shows an intensity modulation depth of about 10% and an ultrafast relaxation time of about 3 ps at a pump power of 100 mW due to the quantum confinement effect. To further analyze the recombination mechanisms of the photogenerated carriers, a three-exponential function is used to fit the carrier decay processes, obtaining three different decay channels, originating from free carrier recombination, exciton recombination, and trap-assisted recombination, respectively. In addition, the photoconductor changes (∆*σ*) at different pump–probe delay times are also investigated using the Drude-Smith model, and a maximum difference of 600 S/m is obtained at τ_p_ = 0 ps for a pump power of 100 mW. Therefore, these results show that the 2D (PEA)_2_(MA)_2_Pb_3_I_10_ film has potential applications in high-performance tunable and ultrafast THz devices.

## 1. Introduction

In recent years, terahertz (THz) waves have received great attention from various researchers because of their special properties and promising applications in many fields. Currently, THz generators and detectors have been reported and consistently demonstrated, while THz functional devices have faced great challenges due to a lack of the appropriate nature materials [1,2,3]. THz modulators, as key components of THz communication fields, can modulate the amplitude, phase, and polarization of THz waves by exciting active materials to implement different functions [4,5,6]. Traditional semiconductors can achieve a high modulation speed among these active materials with ultrafast optical pumping. To achieve a high modulation depth, however, a high power is required to produce photogenerated carriers, thus significantly restricting their application fields [7]. Recently, three-dimensional (3D) organic–inorganic hybrid halide perovskites have achieved unprecedented and rapid developments in the field of photoelectric devices due to their high absorption efficiency, adjustable energy band, high defect tolerance, and good carrier transport performance [8,9]. However, these conventional 3D halide perovskites are sensitive and unstable to water, light, and heat, meaning that there are high requirements for their preparation and preservation environments, thereby limiting their wide commercial applications [10,11]. For the previously studied CH_3_NH_3_PbI_3_ perovskite, for example, the methylamino cation CH_3_NH_3_^+^ (MA) is extremely soluble in water, and thus readily produces PbI_2_ in the perovskite, causing irreversible chemical damages [11]. 

To solve the above issues, a commonly used method is to replace the organic cation in 3D perovskites with a hydrophobic long-chain organic cation to construct the 2D perovskites, thus enhancing the resistance to water molecules and environmental factors (such as ultraviolet rays and heat), and as a result, greatly improving the stability of the organic–inorganic hybrid perovskites [12,13,14,15,16,17]. Currently, 2D organic–inorganic halide perovskites have been rapidly developed in the field of optoelectronics due to their high stability, tunable photoelectric property, and high quantum efficiency [18,19,20,21]. For example, Karunadasa et al. first prepared a 2D organic–inorganic hybrid perovskite solar cell with an active layer of (PEA)_2_(MA)_2_Pb_3_I_10_, which can still maintain a high efficiency when placed in an environment with a relative humidity of 52% for 46 days [18]. After that, Sargent et al. designed (PEA)_2_(MA)_n−1_Pb_n_I_n+1_ (n > 40) perovskite systems, which can realize a short-circuit current of up to 19.12 mA/cm^−2^ and conversion efficiency of 15.3% [19]. In 2016, Jinwoo et al. used the (PEA)_2_(MA)_n−1_Pb_n_Br_n+1_ (n = 1~4) perovskite as a light-emitting layer to prepare high-efficiency quasi-2D light-emitting diodes (LEDs) with a current efficiency of 4.90 cd/A [20]. In 2021, Xu et al. fabricated highly efficient quasi-2D perovskite light-emitting diodes with a maximum brightness of 35,000 cd/m^2^ and maximum external quantum efficiency (EQE) of 12.4% [21]. Although 2D perovskites have been extensively studied in photoelectric devices, the THz and ultrafast characteristics of the 2D perovskites are seldom reported, limiting the developments and applications of the tunable and ultrafast THz devices based on 2D perovskites [22].

In this paper, we investigated the THz and ultrafast responses of a 2D (PEA)_2_(MA)_2_Pb_3_I_10_ perovskite film to reveal the decay mechanisms of the photogenerated carriers. Firstly, (PEA)_2_(MA)_2_Pb_3_I_10_ perovskite films with n = 3 (PEA content of 50%) were prepared on the quartz substrates by a one-step spin-coating process. Then, the structural and photoelectric properties of the prepared films were characterized by the scanning electron microscope (SEM), X-ray diffraction (XRD), ultraviolet–visible (UV–vis) spectroscopy, and photoluminescence (PL) technologies, respectively. Next, the THz and ultrafast responses were measured using our homemade ultrafast optical pump–THz probe (OPTP) system, obtaining an intensity modulation depth of about 10% and an ultrafast relaxation time of about 3 ps for the pump power of 100 mW. To further discover the recombination mechanisms of the photogenerated carriers, finally, the decay process of the carriers was fitted using a three-exponential formula. Therefore, the prepared 2D (PEA)_2_(MA)_2_Pb_3_I_10_ perovskite has broad application prospects in the design of high-performance tunable and ultrafast THz devices. 

## 2. Structure of 2D Perovskites

**Figure 1:**
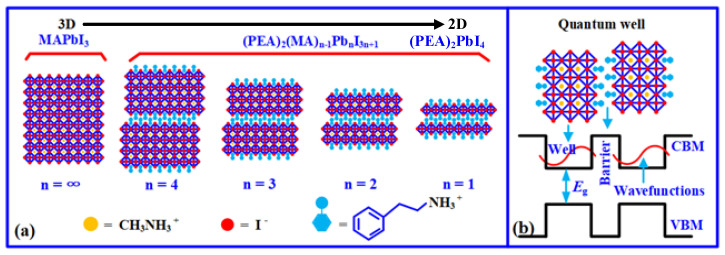
Structural schematic of the perovskites: (**a**) evolution process of perovskites from 3D to 2D and (**b**) multi-quantum well structure.

Generally, 2D organic–inorganic hybrid perovskites are expressed as (RNH_3_)_2_A_n−1_M_n_X_3n+1_ (n = 1, 2, 3, 4……), where R is the organic group, and *n* is the number of the stacked diagonal octahedral layers, namely the number of organic layers. When *n* is equal to 1 or a finite integer, the (RNH_3_)_2_A_n−1_MnX_3n+1_ can be considered as a pure-2D or quasi-2D structure, while becoming a 3D structure as n = ∞, as shown in Figure 1. Thus, 2D organic–inorganic perovskites can be constructed by replacing the A in 3D structures with the hydrophobic organic macromolecules [20]. Figure 1a shows the structural schematics of the (PEA)_2_(MA)_n−1_Pb_n_I_3n+1_ perovskites with different PEA percentages, in which the organic and inorganic layers are interbedded with each other, resulting in the formation of a natural multi-quantum well structure, as shown in Figure 1b. Therefore, the 2D organic–inorganic perovskites not only have excellent environmental stability due to the existence of hydrophobic molecules resisting moisture, light, and heat, but they also have a large exciton binding energy and excellent photoelectric properties due to the presence of the quantum well structure [18,23]. 

## 3. Fabrications and Characterization of 2D Perovskite Films

**Figure 2:**
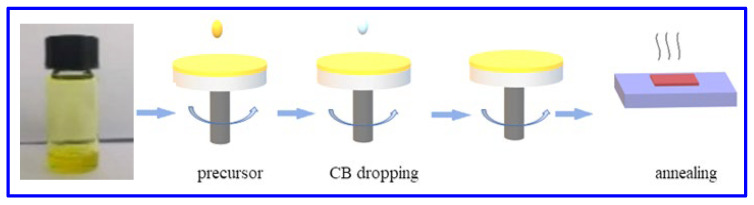
Fabrication process for (PEA)_2_(MA)_2_Pb_3_I_10_ perovskite film.

To obtain high-quality 2D perovskite films, it is very important to follow the fabrication procedures to ensure the growth of high-quality grains. Here, a one-step spin-coating process is applied to fabricate 2D perovskite films. Figure 2 shows the fabrication process of the 2D (PEA)_2_(MA)_2_Pb_3_I_10_ perovskite film. The detailed fabrication steps are as follows: firstly, the MAI and PEAI powders are dissolved in a DMF solution at a ratio of 1:1 to form a precursor solution. Then, the precursor solution is deposited on the surface of a 1 cm × 1 cm quartz substrate by the one-step spin-coating process with two consecutive stages (the first stage at 1000 r/min for 10 s, and the second with 3000 r/m for 50 s). Moreover, in the 30 s of the second spin-coating stage, the antisolvent (chlorobenzene) is continuously dripped onto the substrate to quickly form a uniform film. After spin-coating, the fabricated samples are transferred to a hot plate and annealed at 100 °C for 10 min to remove the residual solvents and transit the intermediate solvate phase into the perovskite, finally forming homogeneous 2D (PEA)_2_(MA)_2_Pb_3_I_10_ perovskite films. All of the fabrication steps are implemented inside a nitrogen-filled glove box at room temperature.

**Figure 3:**
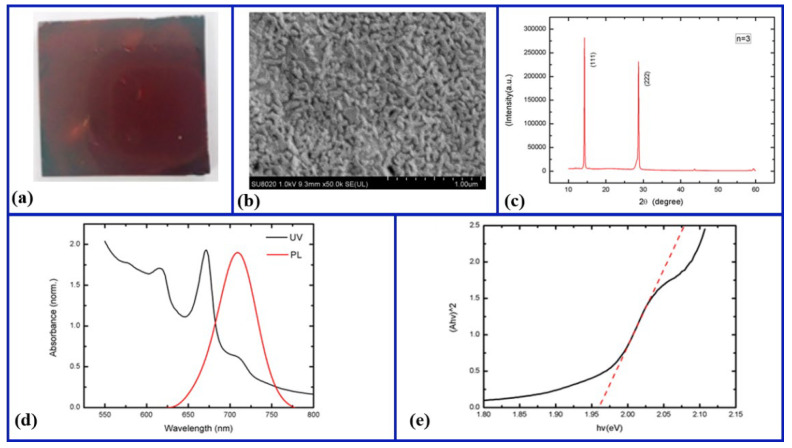
Structural morphologies and optical properties of (PEA)_2_(MA)_2_Pb_3_I_10_ perovskite: (**a**) optical microscope image, (**b**) SEM image, (**c**) XRD patterm, (**d**) absorption profile and PL spectrum, and (**e**) bandgap extraction.

To examine the structural morphologies and optical properties of the as-fabricated perovskite films, next, we caried out different measurements and characterizations using SEM, XRD, UV–vis, and PL technologies, as shown in Figure 3. Figure 3a shows an optical microscope image of the 2D (PEA)_2_(MA)_2_Pb_3_I_10_ perovskite film, which indicates that the perovskite film is very flat and dense owing to the uniform distributions of the precursor solution on the substrate. Figure 3b displays a top-view SEM micrograph of the 2D (PEA)_2_(MA)_2_Pb_3_I_10_ perovskite film, in which the perovskite appears to have nanorod-like crystalline features at the length scale of hundreds of nanometers and a few pinholes due to tight contact between the grain boundaries. Figure 3c shows an XRD pattern of the perovskite film. In the XRD pattern, there are two diffraction peaks at 14.2° and 28.72°, corresponding to the (111) and (222) crystal planes, respectively, which is consistent with the previously reported crystal plane positions [24]. Moreover, no characteristic peaks associated with PbI_2_ or other redundant phases are observed, suggesting that the fabricated films were fully generated with a high crystallinity. Figure 3d presents the UV–vis absorption and PL emission spectra of the as−grown perovskite film. The PL emission spectrum shows only a PL peak with a full width at half maximum (FWHM) of 47 nm centered near 710 nm, while the absorption spectrum shows that the perovskite film has a broad absorption spectrum with three absorption peaks between 600 and 750 nm, indicating a bandgap of ~1.96 eV, as shown in Figure 3e. Moreover, these exciton absorption peaks mainly arise from the low n−member perovskite compounds, demonstrating the presence of 2D perovskites as well as multiphase [25]. In addition, non-zero values of absorbance below the absorption onset are also observed, which is consistent with the fact that the rougher film morphology of perovskites results in a large amount of scattering. Therefore, the above measurement results demonstrate that the as-grown perovskite is a 2D layer structure. 

## 4. Terahertz and Ultrafast Responses of 2D Perovskite Films

**Figure 4:**
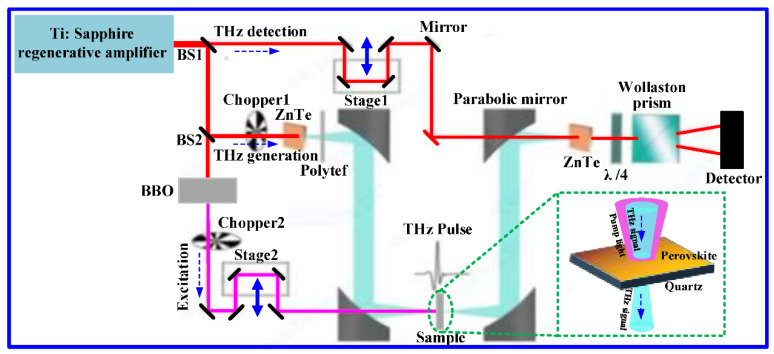
Schematic diagram of the homemade OPTP measurement system.

As is well-known, THz waves are very sensitive to changes in external circumstances. When the semiconductor films are pumped by external light, for example, the THz waves passing through them would be modulated due to the existence of photogenerated carriers. Moreover, the more photogenerated carriers, the greater the modulation depth of the device [26]. Thus, the application prospects of the materials can be further developed according to their modulation depths and speeds. A 2D (PEA)_2_(MA)_2_Pb_3_I_10_ perovskite is a direct bandgap semiconductor and can absorb the photons with energy larger than its bandgap width when pumped by the external light, producing great photogenerated carriers due to the transition of the electrons as a result. To evaluate the THz modulation depth and ultrafast characteristics of the fabricated 2D perovskite films, we set up an OPTP system mainly consisting of the ZnTe crystal-based THz generation and detection beams and an optical pump beam photoexciting the samples, as shown in Figure 4. 

In this homemade system, an amplified Ti–sapphire laser with a pulse duration of 90 ps, wavelength of 800 nm, spectral width of 28 nm, and repetition rate of 75 MHz is used as the optical source for the generation and detection of the THz signal and the photoexcitation of the samples [27]. The laser output beam is split into three beams, where one beam is employed to excite the ZnTe crystal to generate a THz pulse, the second beam is used to detect the THz pulse via free-space electro–optic sampling in a ZnTe crystal, and the third part is used to generate a frequency-doubled 400 nm pump pulse using a barium borate (BBO) crystal to excite the 2D perovskite sample. Moreover, the 400 nm pump beam has an energy of 3.1 eV higher than the bandgap of 2D perovskites (1.96 eV), which can photoinduced free carriers and excitons. In addition, the diameter of the pump beam is 5 mm, which is larger than the diameter (2 mm) of the focused THz beam to ensure uniform photoexcitation. Thus, the ultrafast response measurements in the OPTP system are carried out by varying the delay time (τ_p_) between pump and detection beams using a translational delay stage, while terahertz time-domain spectrum measurements are performed by fixing the pump pulse at the desired position and sampling the THz pulse using another translational delay stage. As a result, the frequency-dependent terahertz spectroscopy can be obtained after the Fourier transform [28].

**Figure 5:**
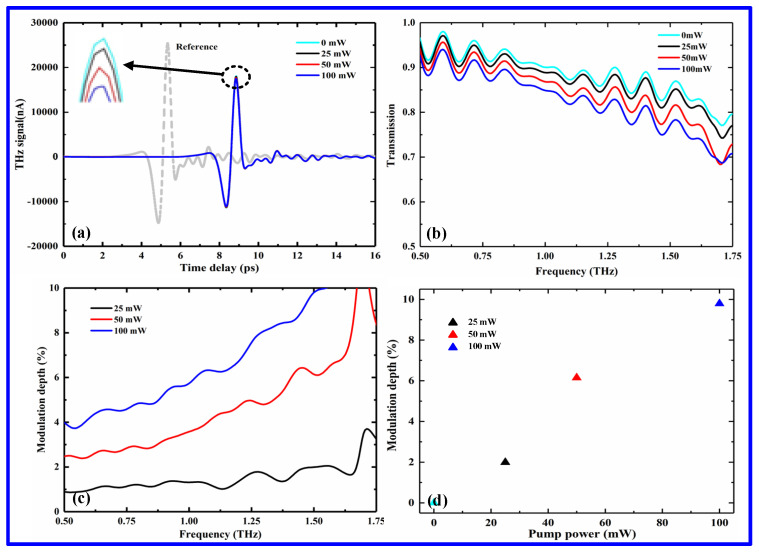
Terahertz performances of the fabricated (PEA)_2_(MA)_2_Pb_3_I_10_ perovskite films at different irradiation powers of 25 mW, 50 mW, and 100 mW: (**a**) terahertz time−domain transmission spectra, in which the inset shows the magnification values at the peak, and the dashed line represents the time-domain transmission spectrum of the reference substrate without perovskite film; (**b**) terahertz frequency-domain transmission spectra; (**c**) modulation depth over the broadband range of 0.5–1.75 THz; and (**d**) modulation depth of different pump powers at 1.5 THz.

To examine the THz modulation ability of the fabricated perovskite films, next, the THz time–domain spectra across the sample, fabricated onto a quartz substrate with a thickness of 2 mm, are measured using our OPTP system at different pump powers. The measurement results are shown in Figure 5a, where the gray dotted line is the reference value of the quartz substrate without the perovskite film. There is a significant time delay between the reference and the sample, demonstrating that the perovskite film were fabricated on the substrate. Figure 5b shows the normalized THz transmission spectra of the fabricated sample for different laser excitations, clearly observing a gradual reduction in the THz transmission with the increase in pump power. The change in the transmission spectra can be attributed to the generation of free carriers in the perovskites. For example, in the absence of a pump beam, the carriers in the perovskite are in the thermal balance state, and there is no observed split in the energy level of the perovskite. In this case, the perovskite has a few carriers that can freely move, and thus obtains a transmission intensity of about 90% at 1 THz. Once the perovskite film is pumped with different pump powers. However, the carriers are generated in the perovskite, breaking the thermal equilibrium state of the perovskite. By further increasing the pump power, the numbers of electrons and holes in the conductivity and valence bands of the perovskite can be gradually increased, leading to a reduction in terahertz transmission intensity, achieving a THz intensity change of nearly 5% at 1THz for a pump power of 100 mW. 

To further assess the modulation performance of the (PEA)_2_(MA)_2_Pb_3_I_10_ perovskite film, a modulation depth (*MD*) was introduced to quantify the modulation ability of the perovskite film at different irradiation powers, which can be expressed as

(1) $$MD=\frac{\int P_{laser-off}(\omega )d\omega -\int P_{laser-on}(\omega )d\omega }{P_{laser-off}(\omega )}$$

where *P_laser_*_-*on*_(*ω*) and *P_laser_*_-*off*_(*ω*) represent the THz amplitudes as the laser pump beam is turned on and off, respectively [29]. As shown in Figure 5c,d, the intensity modulation depth of the fabricated 2D (PEA)_2_(MA)_2_Pb_3_I_10_ perovskite films is gradually enhanced with the increase in pump power, showing a linear increase for different pump powers. The maximum intensity MD is found to be about 10% at 1.5 THz for the pump power of 100 mW. Moreover, the lower MD can be further improved by increasing the pump power. Therefore, the (PEA)_2_(MA)_2_Pb_3_I_10_ perovskite is demonstrated as a promising material that can implement a highly efficient THz modulation.

**Figure 6:**
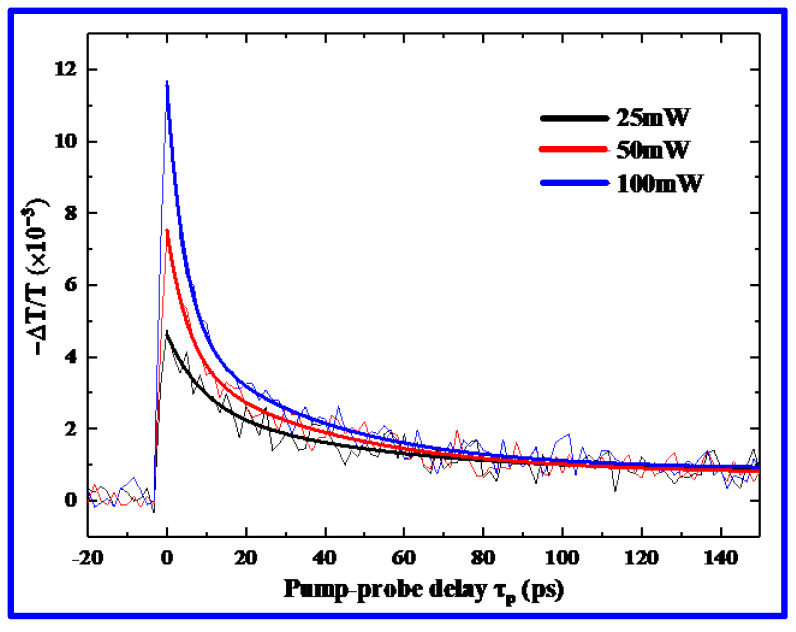
Carrier transient dynamics of 2D (PEA)_2_(MA)_2_Pb_3_I_10_ perovskite film at different pump powers (The thin and thick solid curves correspond to the measured and fitted results).

Next, to explore the ultrafast relaxation response of the photogenerated carriers, the homemade OPTP system is used to monitor the dynamic decay process of the photogenerated carriers by varying the relative delay time (*τ*_p_) between the pump and detection beams. In this experiment, the sample is excited by a femtosecond laser beam with a 400 nm wavelength at the normal incidence, and the probing THz electric field vector is parallel to the plane of the surface of the sample [30]. Following photoexcitation, generally, the relative change in the THz electric field is proportional to the photoinduced conductivity of the pumped material due to the presence of free charges. Thus, the dynamics of charge carriers are manifested as the photoinduced THz transmission changes (−ΔT/T_0_) in the samples at the peak of the THz pulse as a function of pump–probe delay. Figure 6 shows the transient THz transmission dynamics following the photoexcitation of the prepared 2D (PEA)_2_(MA)_2_Pb_3_I_10_ perovskite film for a range of pump powers. In these experimental results, it is noted that the nonequilibrium carriers relax at ultrafast speeds, fully recovering the equilibrium state within a dozen picosecond time scale for the pump power of 25 mW. Moreover, the fast relaxation becomes increasingly significant with the increase in the pump power, indicating that the 2D (PEA)_2_(MA)_2_Pb_3_I_10_ perovskite has potential application prospects in ultrafast THz devices. This ultrafast phenomenon can be attributed to the inherent multi-quantum well structure in the 2D perovskites. 

To further understand the ultrafast relaxation dynamics of the 2D (PEA)_2_(MA)_2_Pb_3_I_10_ perovskite, a triexponential decay function is used to fit the measured THz transient dynamics at different pump powers, extracting the carry lifetimes of the ultrafast processes to discover the recombination channels of the photoexcited free carriers and excitons. Thus, the triexponential decay function is given by the following express [31]

(2) $$f\left(t\right)=A_{1}e^{-\frac{t}{\tau_{1}}}+A_{2}e^{-\frac{t}{\tau_{2}}}+A_{3}e^{-\frac{t}{\tau_{3}}}$$

where *τ*_1_, *τ*_2_, and *τ*_3_ are the lifetimes of different relaxation processes, respectively. *A*_1_, *A*_2_, and *A*_3_ are the corresponding coefficients of each lifetime component, which determine the weights of the decaying and nondecaying components separately. By fitting the measured THz transient changes obtained using our OPTP system, the lifetimes are extracted for different pump powers, as summarized in Table 1. As observed in Table 1, the lifetimes of the three components are *τ*_1_ ~ 10 ps, *τ*_2_ ~ 33 ps, and *τ*_3_ ~ 2 ns for a pump power of 25 mW, respectively. With the increase in the pump power, the initial fast relaxation process becomes faster, while the slow process becomes slower, obtaining *τ*_1_ ~ 3 ps, *τ*_2_ ~ 18 ps, and *τ*_3_ ~ 6 ns for the pump power of 100 mW as a result. These results indicate that such a decay process usually involves three recombination pathways: monomolecular recombination, bimolecular recombination, and Auger recombination [32]. At a lower pump power, the photogenerated carrier relaxations are dominated by the monomolecular decay (*τ*_3_), corresponding to the slow process, whereas at a higher pump power, the recombination channels are dominated by the bimolecular decay (*τ*_2_) and Auger decay (*τ*_1_), corresponding to the fast process. Thus, the monomolecular decay component observed at a lower pump power arises most likely from trap-assisted recombinations, depending on the trap cross-section, energetic depth, density, and distribution. For higher pump power, the bimolecular decay originates from the overlaps of electron and hole wavefunctions, while the Auger process results from the exciton–exciton scatterings, where the excitons are localized inside the QW structures, providing an additional channel for the fast relaxation of free carriers [30]. 

To gain further insights into the ultrafast relaxation behaviors with three exponential decay components (*τ*_1_, *τ*_2_, and *τ*_3_), the spectral dispersions of the THz photoinduced conductivity (Δσ) at different pump–probe delay times (*τ*_p_) were derived from the measured THz transmission transient dynamics using the following expression [33]:

(3) $$\Delta \sigma \left(\omega ,\tau_{p}\right)\approx -\frac{n+1}{Z_{0}}\;\frac{\Delta T\left(\omega ,\tau_{p}\right)}{T\left(\omega ,\tau_{p}\right)}/d\;\left[S/m\right]$$

where *n* is the refractive index of the quartz substrate, whose value is 1.95 at the terahertz range; *Z*_0_ = 377 Ω is the impedance of free space; and *d* is the thickness of the perovskite film.

**Figure 7:**
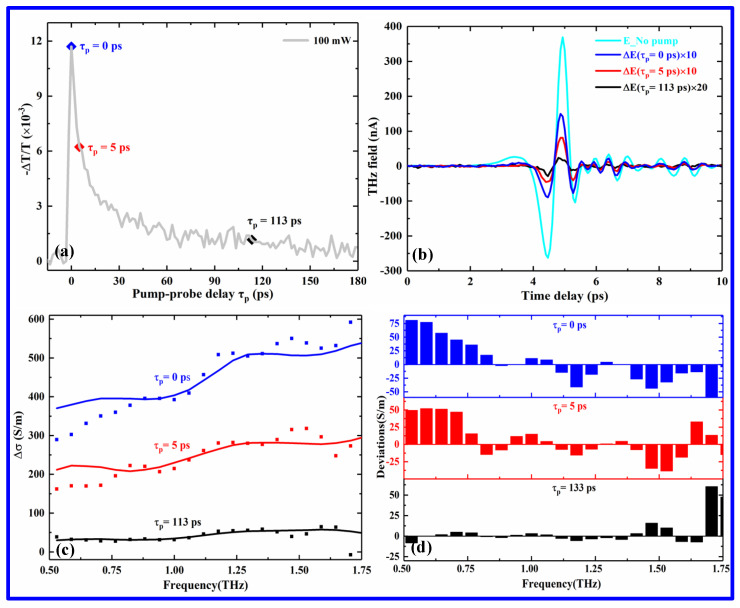
Changes in the THz transmission field, and photoinduced conductivity of the (PEA)_2_(MA)_2_Pb_3_I_10_ film at a pump power of 100 mW: (**a**) THz transmission change at different pump−probe delay times, (**b**) THz field change at *τ*_p_ = 0, 5 and 113.3 ps, (**c**) THz photoinduced conductivity changes at *τ*_p_ = 0, 5 and 113.3 ps, and (**d**) deviations between the extracted and fitted photoinduced conductivities.

Figure 7a shows a typical trace of THz transmission for the pump power of 100 mW, which reveals the transient dynamics of free carriers and excitons in the 2D perovskite film, as discussed above. Figure 7b displays the THz electric field changes at different pump–probe delay times, as shown in Figure 7a (blue, red, and black solid curves correspond to *τ*_p_ of 0, 5, and 113 ps, respectively, in which the ∆*E* is enlarged by over ten times for clarity), while Figure 7c displays the changes in the THz photoconductivity extracted using the corresponding THz electric field changes, which is shown by scattered points. It is noted that the variations of the THz intensity and photoconductivity are increasingly weakened with the increase in the pump–probe delay time. For example, at *τ*_p_ = 0 ps, the photogenerated carriers start to decay and relax quickly, and THz intensity and conductivity exhibit maximal changes due to the existence of abundant photogenerated carriers. As *τ*_p_ is increased from 0 to 5.0 ps, the change in THz intensity and conductivity is gradually decreased due to the recombination of the photogenerated carriers. At *τ*_p_ = 113 ps, however, the system is almost restored to the initial balanced state, and the photogenerated carriers have been fully recombined, leading to the minimal change of the THz intensity and conductivity. Such a change trend can be attributed to the change in the photoinduced free carry density [34]. In addition, the Drude−Smith model is used to further fit the extracted photoinduced THz conductivities (solid curves of Figure 7c), and the corresponding deviations are shown in Figure 7d. It is noticed that in the early process, the extracted photoinduced THz conductivities show a considerable disparity from the Drude−Smith model due to the complicated THz responses, as shown in the top row of Figure 7d. Such a remarked deviation can result from the contributions of both the charge carrier transport and exciton–phonon scattering [35]. For the slow decay process (*τ*_p_ = 113 ps), however, the extracted values agree well with the fitting values (see the bottom row of Figure 7d), indicating a primary contribution from the defect trapping process [36]. 

## 5. Conclusions

In summary, we prepared the (PEA)_2_(MA)_2_Pb_3_I_10_ perovskite films by a one-step spin-coating process and characterized them by different measuring methods. The SEM, XRD, UV–vis, and PL measurements demonstrate that the as-grown (PEA)_2_(MA)_2_Pb_3_I_10_ perovskite films are a 2D layer structure. The OPTP measurements show that the 2D (PEA)_2_(MA)_2_Pb_3_I_10_ perovskite film can achieve an MD of up to 10% at 1.5 THz and an ultrafast relaxation time of about 3ps at an illumination power of 100 mW. Moreover, the fitting results obtained by a three-exponential function reveal that the decay mechanism involves the monomolecular, bimolecular, and Auger recombination processes, corresponding to the free carrier relaxation, exciton recombination, and trap-assisted recombination, respectively. In addition, the changes in the photogenerated conductivity at different pump–probe delay times were extracted and fitted using the measured THz transient dynamics and the Drude-Smith model, respectively, obtaining a maximum change of 600 S/m at *τ*_p_ = 0 ps. Therefore, these results show that the 2D (PEA)_2_(MA)_2_Pb_3_I_10_ film has potential applications in high-performance tunable and ultrafast THz devices.

## Figures and Tables

**Table 1:** Extracted lifetimes of different pump powers by the triexponential fitting.

	Power	25 mW	50 mW	100 mW
Lifetime				
*τ*_1_ (ps)		10 ± 0.2	6 ± 0.2	3 ± 0.1
*τ*_2_ (ps)		32 ± 0.8	30 ± 0.9	18 ± 1.2
*τ*_3_ (ps)		1830 ± 30	3341 ± 50	5854 ± 50

## Data Availability

Data is contained within the article.
